# Supplementary material for: The A domain of clonal complex 1-type fibronectin binding protein B promotes adherence and biofilm formation in Staphylococcus aureus
Source: Microbiology (Reading). 2023 Jun 16;169(6):001348. doi: 10.1099/mic.0.001348 (PMC10333788; doi:10.1099/mic.0.001348)
Supplement: Supplementary material 1 [file mic-169-1348-s001.pdf]

Supplementary Information

The A domain of clonal complex 1-type fibronectin binding protein B promotes adherence and biofilm formation in *Staphylococcus aureus*.

Sara R. Henderson, and Joan A. Geoghegan

Institute of Microbiology and Infection, College of Medical and Dental Sciences, University of Birmingham, Edgbaston, B15 2TT, UK.

Corresponding author: Joan A. Geoghegan, j.geoghegan@bham.ac.uk

Table S1. Bacterial Strains

Strain	Description	Reference
E. coli XL1-Blue	Host for cloning, *recA1 endA1 gyrA96 thi-1 hsdR17 supE44 relA1 lac*	Stratagene
E. coli IM01B	Plasmid propergation. SA08BΩP_{N25-} *hsdS* (CC1-1)	(1)
S. aureus SH1000	Derivative of strain 8325-4 with a repaired rsbU gene	(2)
S. aureus SH1000 *clfAclfBfnbAfnbB*	Adhesion deficient mutant. ClfA, ClfB, FnBPA and FnBPB are disrupted	(3)
E. coli Topp3	Strain deficient in proteases for expression of recombinant proteins. Rifr , [F’proAB+	Stratagene

Table S2. Primers

ClfA_BB_rev	CTGTAGTATTGTTTTGTTCCGATGCTGCATCTGCTTCTTTACTGCTGAG
ClfA_BB_for	TAATAACGCTCAAGGCGACGGTAAAGATTCAGATTCTGACCCAGGTTCA
FnBPB_N1_F	GCATCGGAACAAAACAATACTACAGTAG
FnBPB_N3_R	TTTACCGTCGCCTTGAGCGTTATTAGAGTA
FnBPB_F1_N312A_F314A	GGCTGAATTAAACTTA**GCT**TTG**GCT**ATTGATCCAAC
FnBPB_R1_N312A_F314A	CGTAACTGTAGTTGGATCAAT**AGC**CAA**AGC**TAAGTT
FnBPB_F1_N312A	GGCTGAATTAAACTTA**GCT**TTG**TTT**ATTGATCCAAC
FnBPB_R1_N312A	CGTAACTGTAGTTGGATCAAT**AAA**CAA**AGC**TAAGTT
FnBPB_F1_F314A	GGCTGAATTAAACTTA**AAT**TTG**GCT**ATTGATCCAAC
FnBPB_R1_F314A	CGTAACTGTAGTTGGATCAAT**AGC**CAA**ATT**TAAGTT

Bold = Nucleotide Changes; Underline = complimentary overhangs.

Table S3. Plasmids

Plasmid	Description	Reference
pALC2073	Tetracycline inducible expression plasmid. Cm^r resistance.	(4)
pALC2073::*fnbB*	pALC2073 carrying the *fnbB* gene from CC1 *S. aureus*. Cm^r	(5)
pALC2073::*clfA*	pALC2073 carrying the *clfA* gene from CC8 *S. aureus*. Cm^r	(6)
pALC2073::*fnbB*(A)	pALC2073 carrying the *clfA* gene from CC8 *S. aureus*, where the *clfA* A-domain is replaced with the sequence of the CC1 type *fnbB* A domain. Cm^r	This study
pALC2073::*fnbB*(A)_N277A	pALC2073 carrying the *clfA* gene from CC8 *S. aureus*, where the *clfA* A-domain is replaced with the sequence of the CC1 type *fnbB* A domain with mutations resulting in the N277A substitution. Cm^r	This study
pALC2073::*fnbB*(A)_F279A	pALC2073 carrying the *clfA* gene from CC8 *S. aureus*, where the *clfA* A-domain is replaced with the sequence of the CC1 type *fnbB* A domain with mutations resulting in the F279A substitution. Cm^r	This study

pALC2073::*fnbB*(A)_N277A+F279A	pALC2073 carrying the *clfA* gene from CC8 *S. aureus*, where the *clfA* A-domain is replaced with the sequence of the CC1 type *fnbB* A domain with mutations resulting in both N277A and F279A substitutions. Cm^r	This study
pGEX-4T2	*E. coli* vector for the expression of glutathione S-transferase tagged recombinant proteins. Amp^r	GE Lifesciences
pGEX-4T2::CDSNΔCT	pGEX-4T2::CDSN derivative where DNA encoding residues G376-G76 have been deleted. Amp^r	(5)
pQE30::*fnbB*N2N3	pQE30 carrying DNA encoding CC1-type FnBPB N2N3 subdomains. Amp^r	(7)
pQE30::*fnbB*N2N3_N277A+F279A	pQE30 carrying DNA encoding CC1-type FnBPB N2N3 subdomains, with mutations resulting in both N277A and F279A substitutions. Amp^r	This study

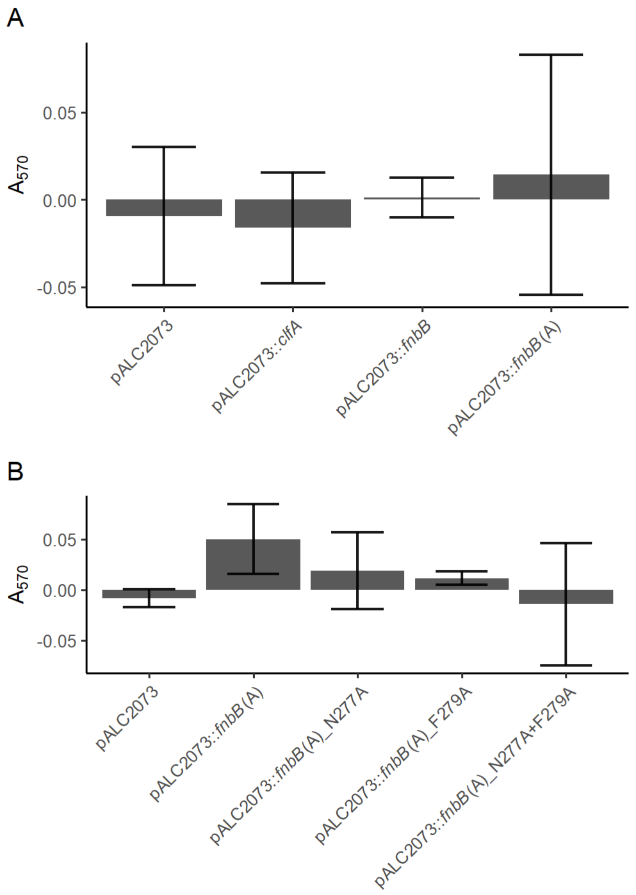

Fig. S1: Adhesion of SH1000 *clfAclfBfnbAfnbB* to GST (300 nM). A) Data corresponds to the strains presented in figure 1 (A) and figure 2 (B). Data is presented as the mean of a minimum of 3 biological replicates, error bars represent 1 Standard deviation from the mean. No statistical significance was determined by 1-way ANOVA with post-hoc Tukey's honest significance test.

A

```
FnBPA(CC8)       ASEQKTTTVEENGNSATDNKTSETQTTATNVNHIEETQSYNATVTEQPSNATQVTTEEAP 96
FnBPB(CC1)       ASEQNNTTVEESGSSATERKASETQTTTNNVNTIDETQSYSATSTEQPSQSTQVTTEEAP 96
FnBPB(CC8)       ASEQNNTTVEESGSSATESKASETQTTTNNVNTIDETQSYSATSTEQPSQSTQVTTEEAP 96
                 ****:.*****.*.***: *:******:.*** *:*****.** *****::*********

FnBPA(CC8)       KAVQAPQTAQPANIETVKEEVVKEEAKPQVKETTQSQDNSGDQRQVDLTPKKATQNQVAE 156
FnBPB(CC1)       TTVQAPKVETS---------------------------------RVDLPSEKVADKETTG 123
FnBPB(CC8)       KTVQAPKVETS---------------------------------RVDLPSEKVADKETTG 123
                 .:****:.                                    :***  :*.::::.: 

FnBPA(CC8)       TQVEVAQPRTASESKPRVTRSADVAEAKE--ASNAKVETGTDVTSKVTVEIGS-IEGHN- 212
FnBPB(CC1)       TQVDTAQPSNVSEIKPRMKRSTDVTAVTEKEVVEEAKATGTDVTSKVEVEEGSEIVGHNN 183
FnBPB(CC8)       TQVDIAQPSNVSEIKPRMKRSTDVTAVAEKEVVEETKATGTDVTNKVEVEEGSEIVGHKQ 183
                 ***: ***  ..** ***:.**:**: . *   . :     ******.** ** ** * **:

FnBPA(CC8)       -NTNKVEPHAGQRAVLKYKLKFENGLHQGDYFDFTLSNNVNTHGVSTARKVPEIKNG-SV 270
FnBPB(CC1)       KETNVVNPHNAERVTLKYKWKFEDGIKPGDYFDFTLSNNVETHGISPLRKVPDIKSKDDN 243
FnBPB(CC8)       -DTNVVNPHNAERVTLKYKWKFGEGIKAGDYFDFTLSDNVETHGISTLRKVPEIKSTDGQ 242
                  :** *:** .:*..**** ** :*:: *********:**:***:*  ****:**.   .

FnBPA(CC8)       VMATGEVLEGGKIRYTFTNDIEDKVDVTAELEINLFIDPKTVQTNGNQTITSTLNEEQTS 330
FnBPB(CC1)       ILAVGKVMDERKIRYTFTDYINNKNNLMAELNLNLFIDPTTVTKQGKQTVEVKLGENKIS 303
FnBPB(CC8)       VMATGEIIGERKVRYTFKEYVQEKKDLTAELSLNLFIDPTTVTQKGNQNVEVKLGETTVS 302
                 ::*.*:::    *:****.: :::* :: ***.:******.**  :*:*.:  .*.*    *

FnBPA(CC8)       KELDVKYKDGIGNY-YANLNGSIETFNKANNRFSHVAFIKPNNGKTTSVTVTGTLMKGSN 389
FnBPB(CC1)       KEFDIKYLDGVKDNWGVTVNGRITLLDKENSKIHHLAYINPKKSDMTSITINGFFAKGGL 363
FnBPB(CC8)       KIFNIQYLGGVRDNWGVTANGRIDTLNKVDGKFSHFAYMKPNNQSLSSVTVTGQVTKGNK 362
                 * ::::* .*: :    .. ** *  ::* :.:: *.*:::*:: . :*:*:.* . **.

FnBPA(CC8)       QNGNQPKVRIFEYLGNNEDIAKSVYANTTDTSKFKEVTSNMSGNLNLQNNGSYSLNIENL 449
FnBPB(CC1)       YTGNVPTVKVYEYLRS-DELPESVYANTNDQEKFKDVTNDMSDKLTLSENGSYKLTLDAL 422
FnBPB(CC8)       PGVNNPTVKVYKHIGS-DDLAESVYAKLDDVSKFEDVTDNMSL--DFDTNGGYSLNFNNL 419
                    * *.*:::::: . ::: :****:  * .**::**.:**    :. **.*.*.:: *

FnBPA(CC8)       D--KTYVVHYDGEYLNGTDEVDFRTQMVGHPEQLYKYYYDRGYTLTWDNGLVLYSNKANG 507
FnBPB(CC1)       N-KKSYVVSFEGKYNENDKELLFRTNLHGYHAN-YGYYYYYPVSLTWDNGVAFYSNNAQG 480
FnBPB(CC8)       DQSKNYVIKYEGYYDSNASNLEFQTHLFGY------YNYYYTSNLTWKNGVAFYSNNAQG 473
                 :  *.**: ::* * .. .:: *:*:: *:       * *    .***.**:.:***:*:*

FnBPA(CC8)       NEK--     510
FnBPB(CC1)       DGKDK     485
FnBPB(CC8)       DGK--     476
```

B

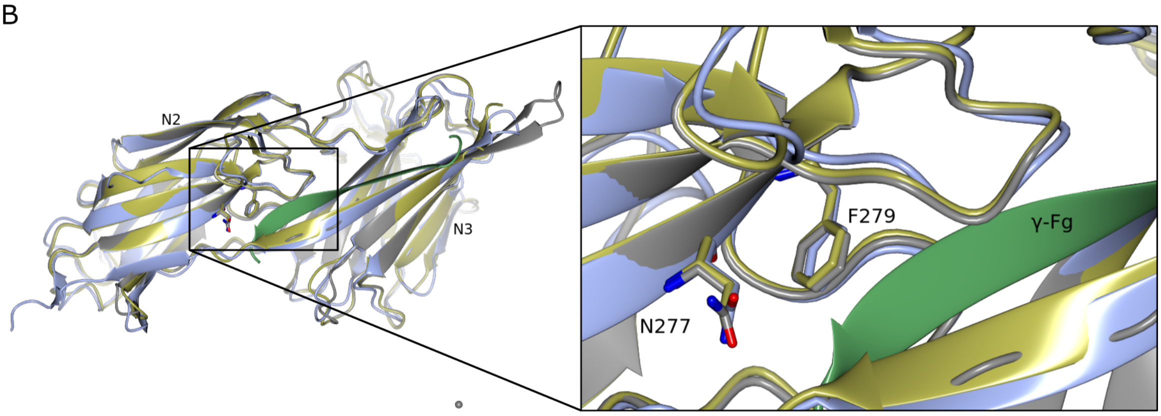

Fig. S2. A) Sequence alignment of the A-region comprising of the N1N2N3 domains of FnBPB from clonal complex 1 (AD08), clonal complex 8 (USA300) and FnBPA from clonal complex 8 (NCTC8325). The N1 domain is indicated in cyan, N2 in green and

the N3 in yellow. Yellow highlighted residues indicate the position of the N277 and F279 residues in CC1 type FnBPB. B) Structural Alignment of the N2N3 domains of FnBPA (Gold) in the presence of the γ-Fibrinogen peptide (Green) (pdb: 4B60) aligned with the AlphaFold-2 prediction of CC8-type FnBPB (Blue) and the Phyre2 prediction of CC1-type FnBPB (Grey). Overview left and detailed view of the ligand binding trench indicating the positions of the N277 and F279 residues in relation to the predicted ligand binding site.

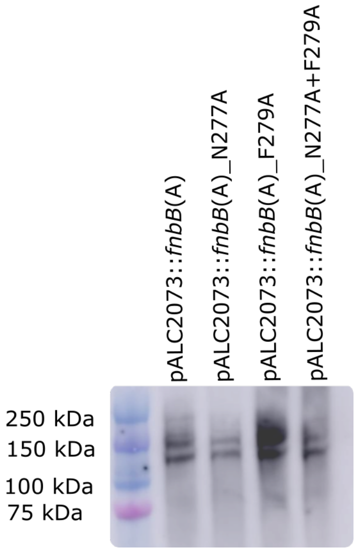

Fig. S3: Expression of the FnBPB A domain in SH1000 *clfAclfBfnbAfnbB* transformed with pALC2073::*fnbB*(A) variant plasmids induced with ATc (300 ng/ml) when cultures reached an OD_{600} = 0.18 and harvested at OD_{600} = 0.35. Washed cells were resuspended to an OD_{600} = 10, and the cell wall anchored proteins extracted and separated on 10% Nu-PAGE Bis-tris gels in MOPS running buffer. Proteins were transferred to PVDF membrane and subsequently blocked with 10% skimmed milk powder. Primary antibody = anti-FnBPB (1:1000). Bound antibody was detected with protein A peroxidase (1:50000).

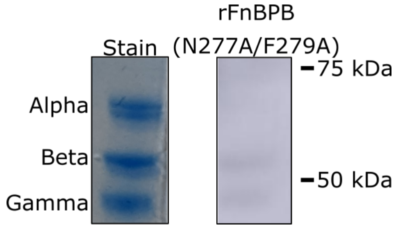

Fig. S4: Recombinant FnBPB N2N3 domains with N277A and F279A substitutions does not bind to the gamma chain of fibrinogen. Fibrinogen was separated on 10% Bis-tris Gels in MOPS running buffer under reducing conditions. The stained portion of the gel was stained to distinguish the positions of the alpha, beta and gamma Fibrinogen chains in decreasing molecular mass. The rFnBPB portion of the gel was transferred to PVDF membrane, blocked with 5% (w/v) BSA (PBS-T) overnight (4°C) and probed with recombinant rFnBPB for 4 hours at room temperature. Bound rFnBPB was detected with HRP-conjugated Anti-His antibody (Roche, 1:1000 in 5% (w/v) BSA PBS-T), and incubated with LumiGlo reagent before chemiluminescent imaging.

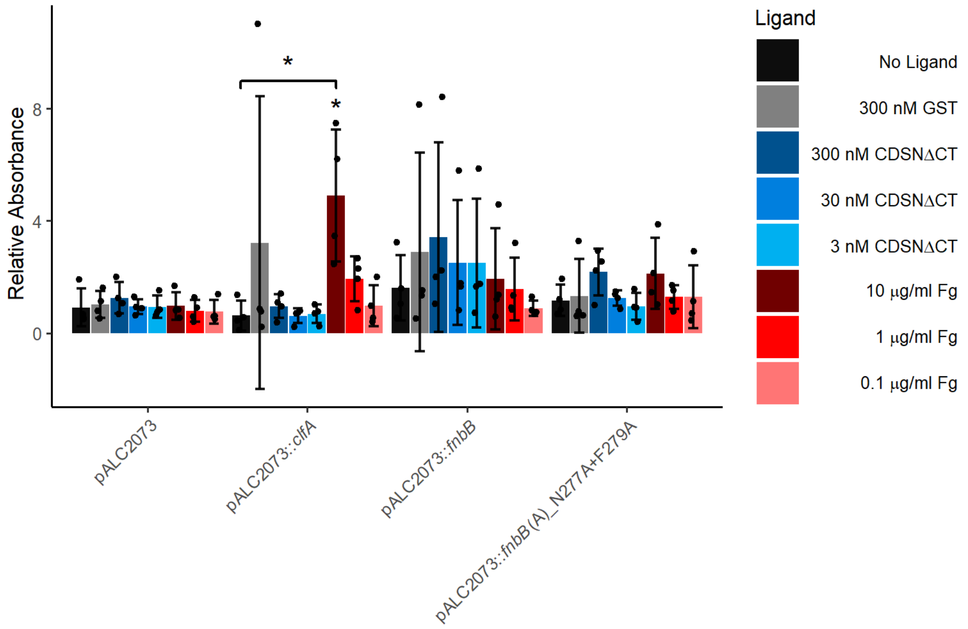

Fig. S5. The impact of FnBPB ligands on biofilm formation. 24 hour biofilms of SH1000 *clfAclfBfnbAfnbB* transformed with pALC2073 and derivatives were grown in TSBG supplemented with anhydrotetracycline (150 ng/ml) with ligands at the specified concentrations. Biofilms were washed and subsequently quantified by staining with crystal violet (1% w/v) solubilised in 70% ethanol. To determine the relative absorbance values the A_{570} values for each biological replicate were divided by those obtained in the absence of a ligand. Data is presented as the mean of 4 biological replicates. Error bars represent 1 standard deviation from the mean. Significance was tested via a 1-way ANOVA test with a Dunnett's Multiple Comparison test comparing each strain to the biofilm formed in the absence of any ligand. *$p<0.05$.

Supplementary references

1. Monk IR, Tree JJ, Howden BP, Stinear TP, Foster TJ. Complete Bypass of Restriction Systems for Major Staphylococcus aureus Lineages. mBio. 2015;6(3):12.
2. Horsburgh MJ, Aish JL, White IJ, Shaw L, Lithgow JK, Foster SJ. sigmaB modulates virulence determinant expression and stress resistance: characterization of a functional rsbU strain derived from Staphylococcus aureus 8325-4. Journal of bacteriology. 2002;184(19):5457-67.
3. O'Neill E, Pozzi C, Houston P, Humphreys H, Robinson DA, Loughman A, et al. A novel Staphylococcus aureus biofilm phenotype mediated by the fibronectin-binding proteins, FnBPA and FnBPB. Journal of bacteriology. 2008;190(11):3835-50.
4. Bateman BT, Donegan NP, Jarry TM, Palma M, Cheung AL. Evaluation of a tetracycline-inducible promoter in Staphylococcus aureus in vitro and in vivo and its application in demonstrating the role of sigB in microcolony formation. Infect Immun. 2001;69(12):7851-7.
5. Towell AM, Feuillie C, Vitry P, Da Costa TM, Mathelie-Guinlet M, Kezic S, et al. Staphylococcus aureus binds to the N-terminal region of corneodesmosin to adhere to the stratum corneum in atopic dermatitis. Proceedings of the National Academy of Sciences of the United States of America. 2021;118(1).
6. McCormack N, Foster TJ, Geoghegan JA. A short sequence within subdomain N1 of region A of the Staphylococcus aureus MSCRAMM clumping factor A is required for export and surface display. Microbiology-Sgm. 2014;160:659-70.
7. Burke FM, Di Poto A, Speziale P, Foster TJ. The A domain of fibronectin-binding protein B of Staphylococcus aureus contains a novel fibronectin binding site. Febs J. 2011;278(13):2359-71.
